# Addressing Challenges in Formal Research on Moribund Heritage Languages: A Path Forward

**DOI:** 10.3389/fpsyg.2021.700126

**Published:** 2021-07-29

**Authors:** Roberta D'Alessandro, David Natvig, Michael T. Putnam

**Affiliations:** ^1^Utrecht Institute of Linguistics, Utrecht University, Utrecht, Netherlands; ^2^Center for Multilingualism in Society Across the Lifespan (MultiLing), University of Oslo, Oslo, Norway; ^3^Pennsylvania State University, University Park, State College, PA, United States; ^4^Centre for Research and Enterprise in Language (CREL), University of Greenwich, London, United Kingdom

**Keywords:** moribund languages, heritage languages, language description, linguistic analysis, theoretical linguistics

## Abstract

The substantial uptick in research on heritage languages over the past three decades has enhanced our understanding of the development of bilingual grammars throughout the lifespan. This interest has been accompanied by a noticeable increase of experimental work, often combined with some degree of formal rigor. Exclusively and predominantly formal research on these languages—especially studies whose empirical focus centers on moribund heritage varieties—occasionally encounters criticism, due primarily to a lack of understanding of the methodology and objectives of this body of research as a whole. The purpose of this positional essay is to once again elucidate with clarity the motivation and importance of formal linguistic research on these languages, providing a fruitful path forward for continued work in this well-established field of linguistic inquiry.

## 1. Introduction

Research focusing on **heritage languages** (HLs) has exponentially increased over the past three decades, contributing exciting and interesting findings, which in turn have enhanced our understanding of the human language faculty and the developmental trajectory of linguistic competence over the course of the lifespan. Arriving at a set definition of the core attributes of a **heritage language speaker** (HS) is notably difficult (Rothman, 2009; Putnam et al., 2018), primarily due to the fact that the status of “being bilingual” is itself fluid and acategorial (Luk and Bialystok, 2013). We agree with Polinsky (2018, p. 6) who asserts that “there is an ebb and flow of language across the lifespan of a [HS],” which further underlies the importance of detailed research on the grammars of these sub-populations of bilinguals. Because HLs are acquired under conditions of variable qualitative and quantitative input when compared with baseline monolinguals (Montrul, 2016), the mental representations associated with these grammars may be different from those of monolinguals, but they are nonetheless complex (to varying degrees).

Our primary goal in this paper is to acknowledge and address the principal challenges and difficulties associated with researching **moribund** (or endangered) HLs and vernaculars. As we discuss in more detail in §2, the majority of moribund HLs are spoken by elderly individuals who usually represent the final or penultimate generation of (highly) proficient speakers of the language and/or dialect. The number of speakers of these HLs is also usually quite small. Due to these unique factors, research conducted in these communities—and the theoretical work that accompanies the collection and interpretation of data and behavioral responses—can differ, sometimes substantially, from other current research endeavors in linguistics. Unfortunately, these differences can be erroneously interpreted by some as fundamentally distinct enough to question the validity of the research conducted on these unique varieties. We feel that the importance of continued research on moribund HLs cannot be overstated if the ultimate goal is to attain the fullest possible understanding of the developmental trajectories of heritage grammars across the lifespan as well as potential universal properties and generalizable tendencies of human language. Furthermore, as has been abundantly shown (Rothman, 2009; Polinsky, 2018; Aalberse et al., 2019, and others), HLs are full and complete grammars, even if they are distinct from the corresponding monolingual grammars. Many structural differences have been observed in HLs which distinguish them from those found in monolinguals (see, e.g., Polinsky, 2018; Montrul and Polinsky, 2021 for an overview of some of the most well-known structural properties an tendencies found in HLs). Nevertheless, HLs are structurally complete, they present no “mutilations” with respect to their monolingual alternates. This body of research to date collectively promotes an image of moribund grammars that, in spite of their endangered status, retain a remarkably high degree of complexity:

…some heritage grammars actually maintain significant complexity in spite of being acquired under conditions of reduced qualitative and quantitative input (Bousquette and Putnam, 2020, p. 189).

Our humble objective is not only to emphasize the importance of this research, but to also provide a path forward that establishes a connection with theoretical work that also connects with historical sociolinguistics (e.g., Geiger and Salmons, 2006; Litty, 2017; Lauersdorf, 2021) (for reasons we discuss below). This manuscript adopts the following structure: In §2 we introduce and critique the common traits of moribund HLs, discussing how these factors contribute to challenges in data elicitation and documentation—especially when compared with current research efforts on HLs in other populations. Our treatment of moribund HLs leads to our discussion of three domains of principal challenges that this research program faces:

- The baseline challenge
- The data elicitation challenge
- The data amount challenge

The **baseline challenge** addresses the dilemma that, at least in most cases, a baseline standard (i.e., the language spoken by speakers from previous generations or some sort of linguistic standard) cannot be established (see also Polinsky, 2018, §8.2 for a treatment of this issue). Second, we confront the **data elicitation challenge**, highlighting that the relatively low number of remaining speakers, their elderly status, and their unfamiliarity with data collection methods and procedures (among other difficulties) often result in data that do not translate easily to received experimental methods. Finally, our discussion of the **data amount challenge** returns us to related debates involving the role of “big data” and statistical significance in linguistic research. In §4 we sketch out the core properties of a research program that addresses these three aforementioned challenges and conclude this manuscript.

## 2. Defining *Moribund* HLs

Speakers of moribund HLs represent a unique, yet equally relevant subgroup of heritage language speakers. In their brief survey of general phonological, morphological, syntactic, and semantic properties of the speech of these individuals, Putnam et al. (2018) advance the claim that their language production does not significantly differ from child and (younger) adult HSs. Bousquette and Putnam (2020) reach similar conclusions in their survey of morphosyntactic properties of Heritage German, providing additional support to the general high degree of functional and structural complexity maintained in the grammars of moribund HLs. These observations notwithstanding, there are general characteristics shared by members of this sub-population of HSs that deserve mentioning, which we explicate in this section.

### 2.1. General Characteristics of Moribund HLs Speakers

The overwhelming majority of moribund HL speakers are elderly; in some cases, they are also 4th or even 5th generation speakers of their vernacular. In other instances, they are 1st or 2nd generation speakers but the last ones to master the language. This is the case, for instance, of the Italo-Romance emigrants to the Americas, as described in Andriani et al. (2021). Those emigrants who left Italy in the 1950 and 1960s almost immediately switched to the host language. This pattern is particularly clear in Argentina, where most 1st generation speakers switched to Spanish immediately after arrival, and, as a result, 2nd and especially 3rd generation speakers of the original Italo-Romance varieties with high proficiency are difficult to find.

The role of bilingualism in connection with aging, the development of grammatical properties across the lifespan, and the interaction with general cognitive abilities (i.e., language processing speed, working memory, inhibitory control, etc.) has been the focus of decades of productive research (Bialystok, 2016; Ivanova et al., 2016; Rossi and Diaz, 2016), pointing toward a potential cognitive “bilingual advantage” that life-long bilinguals hold over their monolingual counterparts. Various domains of grammar show age-related effects, such as lexical retrieval (Goral et al., 2007; Goral, 2014) and the processing involving syntactic complex units (Kemper et al., 2001). The inter- and intra-speaker variation found in old-age language is truly a treasure trove of important linguistic data that we, as a community of scientists, are just beginning to investigate (Pichler et al., 2018). In the special case of moribund HLs, age is indeed a relevant factor that we cannot simply ignore. At the level of linguistic representation (i.e., competence), these populations afford us a unique opportunity to test hypotheses concerning language acquisition and development under quantitatively and qualitatively impoverished circumstances.

A critical question that surfaces at this juncture is whether or not we observe (significant) qualitative and quantitative differences among HSs across the lifespan. A sufficiently exhaustive answer to this question cannot be provided at this time, due to the fact that to date research on “heritage bilinguals” has predominantly focused on child and (younger) adult populations (see, e.g., Putnam et al., 2018 for a review of this argument). This caveat notwithstanding, extant studies focusing primarily (or solely) on the final generation of speakers suggest that by and large moribund HL-grammars retain a high degree of complexity (Bousquette and Putnam, 2020). As a matter of fact, in some instances, these grammars show signs of developmental nuance. Yager et al. (2015) explore how the purported “loss” of dative case markings in three moribund varieties of Heritage German—spoken in Argentina, Kansas, and Wisconsin—have begun to display Differential Object Marking (DOM) in all three communities (to varying degrees). A similar development has been observed in some Italo-Romance varieties in Argentina and Brazil Andriani et al. (2021). Southern Italo-Romance varieties display DOM, which has often been found to be recessive in heritage populations (see, e.g., Montrul, 2004; Montrul and Bowles, 2009; Montrul and Sánchez-Walker, 2013; Montrul et al., 2015; Montrul and Bateman, 2020) and could therefore be expected to be recessive also in heritage Southern-Italian varieties. Recent work by Andriani et al. (2021) show that this previous hypothesis does not hold, and that DOM is instead *extended* to uses that are not attested in the Italian baseline varieties, similar to the findings of Yager et al. (2015).

Leaving interesting details aside for the moment, the aforementioned studies are of primary importance for two reasons: First, they show that nuanced, divergent structures are present even in the final generation of (highly) proficient speakers of a given HL. Second, and of equal importance, they serve as evidence that these divergent forms cannot solely be the product of a lack of use over a prolonged period of time. We maintain that situations such as these can be adduced as further evidence for the complex underlying structure of language advocated in formal approaches.

The presence, and continued emergence, of grammatical innovations serve as counter-evidence to the claims that HL grammars are “incomplete” in some way. Rinke and Flores (2014) express a similar view, supported by their research on clitics in heritage Portuguese. In their own words:

…although the linguistic knowledge of the heritage bilinguals investigated in this study differs from that of monolinguals, it is not “deficient” but “different” and “innovative,” because it is primarily based on the spoken variety of the language and because it promotes linguistic changes which are inherent in the speech of native monolinguals. Cross-linguistic influence seems to play a less important role than generally assumed. (Rinke and Flores, 2014, p. 681).

Another interesting related phenomenon that further supports this point is the emergence of null subjects in structural environments that differ from the baseline (or previous the speech patterns of previous generations, when such data is available for comparison), such as those found in heritage Friulian in Brazil and Argentina. Frasson et al. (2021) show that HSs of Friulian introduce null subjects in topic continuation contexts by dropping the pronominal subject clitic in contexts in which it would be expressed in the baseline. These findings contradict the general assumption that HLs tend to be simplified with respect to the corresponding baseline varieties, as the degree of complexity in some of their structural phenomena is equivalent—if not *higher*—than the corresponding baseline grammar.

### 2.2. Increased or Equal Complexity: American Norwegian

To exemplify the level of complexity and innovation of HL grammars we report here on some general findings on American Norwegian (AmNo). We selected this variety since it has been one of the very first to be targeted by systematic heritage studies, and because it has been described from phonological and morphosyntactic viewpoints. Since Haugen's (1953) influential work on Norwegian in the United States, AmNo has enjoyed a central role in heritage language investigation and theorizing (Aalberse et al., 2019). In the decades following his 1942 field work, American Norwegian-speaking communities underwent a comprehensive shift to English, such that it is exceedingly rare for Norwegian to be acquired and spoken as a heritage language today. As a result, contemporary research on AmNo relies on the language of fewer HSs than nearly 80 years ago. In spite of these challenges, clear patterns emerge from the HL, which themselves deserve description and investigation. We discuss here two examples, one from phonetics/phonology, another from morphosyntax.

For languages such as AmNo that are in sustained contact with English, there has been considerable research into potential influences of English [ɹ] on HL and L2 rhotic patterns, especially for Spanish (Face, 2006; Henriksen, 2015; Amengual, 2016; Kim and Repiso Puigdelliura, 2018). For AmNo, Natvig (2021) finds remarkable stability in the production of the typical tap or trill ([ɾ/r]) variant. Yet, there is a significant increase over time in an English-like [ɹ] approximant in retroflexion environments (see Kristoffersen, 2000, p. 96–97). Although there appears to be a clear influence on the phonetic properties of AmNo /r/ from English, these are restricted to environments that are defined by *Norwegian* phonological processes. Schmid (2011, p. 51–52) finds similar patterns for a German HS, where a uvular [ʁ] occurs in syllable onsets and an English-like approximant in codas, an environment where the German, but not the English, variety has r-deletion. The contact scenarios in both cases appear to have enriched, rather than simplified, the AmNo and German sound systems, but in such a way that their core phonological representations and operations remain intact.

AmNo also evinces a structurally constrained change in morphosyntactic expressions, specifically in the marking of definiteness agreement in noun phrases in what van Baal (2020) refers to as “compositional definitenes” (CD). In the typical pattern, we find both a prenominal determiner, *den* and a suffixed article *-en* that co-occur (along with definiteness agreement on the adjective): *den fin-e bilen* def nice-def car-def.m.sg “the nice car.” Accordingly, the noun phrase, “doubly” marked for definiteness, demonstrates the default CD pattern in modified definite phrases in Norwegian.

van Baal (2020) shows that AmNo speakers produce the same types of modified definite constructions as homeland speakers, but generalize the single-definite marking found for the “inherently definite” modifiers for other adjective types (see Dahl, 2015, p. 235), i.e., with a definite suffix but without a prenominal determiner as in *brun-e hund-ene* brown-def dog-def.pl “the brown dogs.” There is a change relative to homeland Norwegian, but one that is constrained by the inherited Norwegian grammar rather than a direct imposition of English. Although such surface patterns appear to “simplify” the number of overt markers for definiteness in the HL, these bilinguals unquestionably maintain the representations for both preposed (i.e., English) and suffixed (i.e., Norwegian) definite marking in their syntax.

In neither case described here can AmNo be considered as simplified or even less complex than a monolingual Norwegian grammar, although there certainly are structural differences (see Eide and Hjelde, 2015; Johannessen and Larsson, 2015; Larsson and Johannessen, 2015; Lohndal and Westergaard, 2016; Westergaard and Lohndal, 2019; Putnam and Søfteland, 2021). Rather, the patterns and properties of HL grammars, AmNo included, attest to the richness and diversity of language. If we are to pursue knowledge and a deeper understanding of the mechanisms to which that diversity is owed, then moribund HLs must be worthy of investigation and analysis in their own right.

## 3. Addressing “Challenges”

We made the case in the previous section, following similar argumentation laid out by Putnam et al. (2018), for the importance of including data from moribund HLs in research on heritage bilingualism. Taking things a step further, we advance the claim that the failure to do so limits our collective opportunity to achieve a detailed understanding of the development of heritage bilinguals across the entire lifespan. In spite of the importance of obtaining data from these populations, in this section we acknowledge three unique challenges that beset research efforts on moribund HLs. Although researchers who are familiar with these populations will recognize and appreciate these challenges right away, these issues are sometimes not fully understood in the field at large—even by scholars who are active researchers in aspects of bilingualism. Failure to acknowledge and understand these challenges often result in unfavorable assessments of moribund HL research. Here we review these three challenges; namely, (i) the baseline challenge (§3.1), (ii) the data elicitation challenge (§3.2), and (iii) the data amount challenge (§3.3).

### 3.1. The Baseline Challenge

An unfortunate, and patently incorrect, approach to formal research on bilingual grammars is one that analyzes them as if they are somehow incomplete or deformed in some way. Concerning the research on late L2-learners, Ortega (2014) labels such approaches as “deficient-oriented,” i.e., those whose focus centers on the comparative differences between the output forms (production) or comprehension when compared with a “target-like” baseline. Much ink has been spilled on this heated debate concerning the nature and quality of grammatical representations of HLs (Kupisch and Rothman, 2018), and although it is not our intention to contribute to these debates further, an undeniably difficult challenge in researching HLs concerns establishing reliable baseline against which HL-grammars can be compared and measured in some sense. Agreeing in principle with Polinsky (2018, 11), “if we want to understand how heritage language acquisition works, a direct comparison between heritage speakers and monolinguals is not informative”^1^.

Exactly who we ultimately decide to compare heritage bilinguals with may change with respect to the research question of any given study (Polinsky, 2018, §1.2.2), but the general consensus seems to be that the first-generation immigrants of a given speech community were the individuals who provided critical input that shaped the grammars of the next generation (and potentially beyond). But what happens when there is *no* legitimate baseline to be found? This is an all-too-familiar issue for research on moribund HL-populations (which Polinsky, 2018, Ch. 8 refers to as “endangered” heritage speakers). For many under-researched populations, there are no, or very few, extant written or recorded samples of the vernacular upon which one can establish a suitable “baseline.” Speaking in terms of more experimental approaches, under these conditions, we cannot find a valid and reliable “control group,” unless we recruit monolingual speakers, which returns us to an undesirable “deficit-oriented” portrayal of the grammars of HSs.

The baseline challenge is a cover term for a host of sub-issues. For the moment we leave aside the discussion of whether monolingual speakers of a given variety should be considered as a licit control group for the sake of comparison (based on cognitive and/or methodological reasons). Before engaging with this question, we feel it necessary to revisit a handful of issues that, in our opinion, are easily overlooked. Having a firm(er) grasp of these issues is equally important if we wish to improve upon research on HSs from a formal perspective. In the remainder of this section, we turn to the following issues:

- no monolinguals
- no standard variety
- language change

#### 3.1.1. No Monolinguals

Most of the time, in research targeting HL populations we are dealing with languages like Russian, or Korean, or Spanish, for which establishing the baseline grammar or at least the monolingual corresponding grammar can be a somewhat complicated issue which requires requisite knowledge of dialectal variation. However, even rather large heritage varieties, like Catalan, sometimes lack a monolingual counterpart: all speakers of Catalan are bilingual, even in Catalunya. How do we proceed in these cases? We believe that the no monolingual baseline is a false problem, first and foremost because, as stated above, we should, as an accepted default, compare bilinguals with bilinguals, but also because establishing that a variety must be spoken as the only language in one place is ideological to say the least. Multilingualism is the most common situation for humans, while the concept of one language/one territory (with the consequent killing of all minorities) is a typical nineteenth century idea. The one-to-one correspondence of language and country emerged very strongly with European Romanticism. A famous quote by Fichte (1806) exemplifies this perspective:

The first, original, and truly natural boundaries of states are beyond doubt their internal boundaries. Those who speak the same language are joined to each other by a multitude of invisible bonds by nature herself, long before any human art begins; they understand each other and have the power of continuing to make themselves understood more and more clearly; they belong together and are by nature one and an inseparable whole. Such a whole, if it wishes to absorb and mingle with itself any other people of different descent and language, cannot do so without itself becoming confused, in the beginning at any rate, and violently disturbing the even progress of its culture (Fichte, 1806).

The Western tradition of modern linguistic studies, stemming from Europe, has maintained this assumption of the monolingual speaker as “the norm.” This assumption has brought a number of consequences to bear, both on the way we perform linguistic research and on language policy, that are difficult to eradicate. We believe it is time to move forward, and abandon these concepts to the time where they belong.

#### 3.1.2. No Standard

A second important issue that HL researchers often must deal with is *microvariation*: it is not always easy to ascertain whether a phenomenon we see in a HL is the result of change or the preservation of a variant that was originally already there in the language (see e.g., especially Poplack and Levey, 2010 for useful statements concerning the identification of contact-induced language change). Especially when dealing with non-standardized varieties, this issue is crucial. We feel that there is no immediate solution to this problem, especially because there is not always a carefully documented grammar in every place from which the HL originates. However, we feel that this fact is often overlooked, which results in generalizations that are at best inaccurate, and to the false attribution of variation to contact and bilingualism rather than original microvariation.

#### 3.1.3. Language Change

Finally, one additional factor that is consistently overlooked in HL studies is the evolution of grammars, in the sense of language change. The grammars to which we compare the HLs are today's grammars, but the input that HSs received is a grammar from several years, if not centuries, ago, which might be different from the contemporary language spoken in the country of origin. An extreme example of this is heritage Friulian described in Frasson et al. (2021). To begin with, all Friulian speakers in Italy are bilingual (similar to the current day situation in Catalan), so there is no monolingual baseline grammar for comparison (see the previous section). Furthermore, the Friulian emigrants who left Italy in the 1950 and 1960s spoke a language that was not in intense contact with Italian. It was precisely during this time that Italian became in fact the L2 of most Italians and it has since become the dominant, sometimes unique, language around the 1980s (see, e.g., De Mauro, 2011 for a comprehensive overview of the L1 acquisition of Italian). Therefore, comparing the data from people who left Italy in the 1960s with contemporary Friulian is methodologically precarious, as Friulian has evolved independently since. According to Frasson et al. (2021), the best solution is to compare the HSs grammars of today to the Friulian from the time when the emigration wave started (i.e., from the 1950s and 1960s).

Note though that this is not only a problem of minoritized populations, but that it is also a seriously overlooked methodological issue: while there is a lot of discussion on whether the baseline should be monolingual speakers or balanced bilinguals, not many linguists consider that the language spoken by first-generation emigrants can be radically different, in some respects, to the one which is spoken today. Of course, standardized languages may evolve more slowly, but this does not mean that we can take it for granted that the Russian that 1st generation speakers spoke when they left Russia is exactly the same as Russian today. In this respect, even those studies that seem methodologically sound because they have a clear baseline grammar to compare to the HSs show some substantial flaws. The only solution for this is to actually resort to grammars that testify the exact setup of the phenomenon under investigation at the time in which the HSs were severed from their language. We are aware of the fact that this means including an additional factor to control for; however, it is important to raise awareness on the fact that sound historical reconstruction of the sources is also an important issue to be considered, and not just the mono or bilingual status of the baseline speakers.

### 3.2. The Data Elicitation Challenge

Research focusing on moribund HLs—especially any tasks that are online and/or require the manipulation of stimuli—beyond (semi-)structured interviews, elicited narratives, or story-telling can be remarkably challenging for a number of reasons, among which the attitude of HSs with respect to acceptability. In substance, most HSs feel insecure about their recessive language, and tend to dislike expressing judgments or to accept every piece of data, rendering the results obtained from acceptability judgment tasks to be dubious in some instances (see Polinsky, 2018 concerning this issue, to which we will return in §3.2.1)^2^. Furthermore, it is quite hard to find a balanced group of speakers with exactly the same language profiles. Finally, some HSs have issues related to aging which can impact data elicitation (see §2.1). These difficulties often result in lower numbers of participants in studies and a significantly lower number of types and tokens of targeted data forms (which is a challenge we delve more deeply into in §3.3).

Even when the community is not exceedingly small, research involving predominantly elderly populations can be problematic for the researcher and taxing and difficult for the informants. In a recent study Andriani et al. (2021) interviewed 50 heritage Italo-Romance speakers in Brazil, with the average age of their informant being 71. Many elderly speakers were not educated, were not literate in their HL, and therefore could only respond to aural stimuli. However, the researchers report that such stimuli were often also problematic to their informants due to hearing problems (see e.g., Hopp and Putnam's, 2015 for a discussion of how similar challenges impacted participation of elderly informants in aural sentence judgment tasks). Data elicitation then proves more difficult, given that spontaneous speech might not contain all forms of a paradigm.

In summary, eliciting data from small language communities and elderly populations is a noted challenge. Leivada et al. (2019) list 10 challenges in the elicitation of data from small, young, and non-standard languages. Among them, they recognize the difficulty in drawing generalizations from speakers that have different degrees of mastery of the language, especially if the language is not standardized. Furthermore, they acknowledge the limits of generalizations drawn on the basis of a handful non-homogeneous data, especially compared to what is acceptable in experimental research. These issues are easily recognizable: non-standardized varieties have a larger degree of intra-speaker variation. In the case of HSs, this fragmentation is brought to the extreme from the fact that speakers all have different levels of competence in the language, different onset times, different degrees of exposure. Once again, it would be absurd to deny these objective limitations. However, we wish to underline that these limitations are particularly problematic for psycholinguistic studies, but not necessarily for those whose principle aim is to describe the syntactic and phonological structures found in these HLs under investigation.

#### 3.2.1. Asking the Right Questions to the Right People

It is a conceptual, as well as a methodological, mistake to consider that all linguistic studies should be based on and adhere to the methodology commonly applied to psycholinguistic (or “online”) studies, first and foremost because researchers in diverse subfields ask and seek answers to different questions. For research centered on the structure of language, the issues revolve mostly around the limits of syntactic variation; as stated above, to ascertain whether some sentence is grammatical or not in a language one needs to create sometimes complicated, borderline examples, making sure that they are accepted or rejected for the right reasons and not because they are, for instance, pragmatically odd or less frequently attested in usage. This elicitation method, usually referred to as “acceptability judgments” has often been criticized by some and considered inadequate, though repeatedly proven to be perfectly in line with experimental data elicitation (see, e.g., Sprouse et al., 2013). Acceptability, or grammaticality, judgments serve the purpose to explore the boundaries of grammar, and therefore usually contain structures that speakers would not utter spontaneously, but that they would recognize (and evaluate) as possible and licit outputs as regulated by their grammar systems (or not).

Regarding grammaticality judgments, two reflections are in order. First, in principle, if a structure is evaluated to be grammatical, and if it is systematically produced by at least one speaker, this structure deserves further study and investigation. The elicitation of 100 or 10,000 instances of a given phenomenon is not required to validate its existence. Rather, what is at stake here is establishing the categorial status of what is attested, what is not, and how that shapes our understanding of the structure of language (see, e.g., Harbour, 2016, Ch. 1 for a discussion of this delicate distinction in formal linguistic research). Metalinguistic judgments obtained from individual speakers are still held by most linguists as a valid and reliable way of establishing robust claims about the internal structures licensed in individual grammars. Therefore, there is no sensible reason to discard either the data produced by a limited number of speakers or a reduced number of tokens so long as the primary objective is to determine the categorial status of particular forms.

In spite of usefulness and functionality, metalinguistic judgments are a well-known thorn in formal research on HSs, which owes to a number of reasons, most notably due to the non-heterogeneity of the grammars and the general unfamiliarity of the tasks that researchers commonly apply in their elicitation efforts. HSs, as well as other bilinguals, such as L2-learners, often show what Polinsky (2006) refers to as “*yes-bias,”* i.e., the tendency to accept ungrammatical forms in judgment tasks due to a lack of familiar with the task or based on their lack of confidence in their linguistic abilities (see section 3.2). The aforementioned potential shortcomings associated with metalinguistic judgment tasks should not lead us to hastily eschew them from research on HLs; on the contrary, they should just be validated and cross-checked with spontaneous speech and other (structured) production tasks to ensure coherence. Finally, it is worth noting that theoretical syntactic and phonological research is almost never directed at grammar systems in their entirety, but rather the focus is direct toward specific phenomena involving specific parts of the structure.

If HSs have issues with interface phenomena, i.e., syntax-discourse, these should be factored out somehow, or tested separately. Linguists manage to reconstruct historical grammars with no remaining speakers from a few incomplete attestations, and, as we argue here, insights gained from this methodology can be applied to moribund HSs grammars as well, most notably by assembling features that are shared with other speakers and features that belong to individual speakers within the group.

### 3.3. The Data Amount Challenge

The two challenges discussed above, i.e., the challenge trying to identify a suitable baseline couple with the challenge of data elicitation, coalesce into the data amount challenge. It stands to reason that in many instances the shear amount of data elicited from moribund HLs will have weak statistical power and face other related potential shortcomings. Some of the seminal studies of moribund HLs reflect this reality. As a case in point, consider the research of Bullock and Gerfen (2004a), Bullock and Gerfen (2004b), and Bullock and Gerfen (2005) on the phonetic-phonology interface of Frenchville-French, spoken in Frenchville, Pennsylvania. This research is still cited today by a number of handbook-oriented studies and in the research of well-renowned scholars of heritage language phonology and L1 attrition (Celata, 2019; Chang, 2019, 2021; De Leeuw, 2019). What is remarkable about this state of affairs is the fact that this pioneering work carried out by Bullock and Gerfen (2004a), Bullock and Gerfen (2004b), and Bullock and Gerfen (2005) is based on the production of **2 remaining proficient speakers** of this moribund HL. Clearly, under different circumstances more speakers of Frenchville, French would have been sought out, but the importance of these results, and the research conducted in a unique population such as this, has stood the test of time.

Once again, the previous two challenges often conspire to reduce the number of eligible informants for metalinguistic judgment tasks. A clear case of how this challenge plays out in this research is Hopp and Putnam's (2015) investigation of finite verb placement in matrix and subordinate clauses in Moundridge Schweitzer German (MSG). MSG is a moribund Palatinate-based heritage German dialect spoken in South Central Kansas in and around the communities of Moundridge and Pretty Prairie (a.k.a. “Pinch”), Kansas. At the time of their study, the remaining number of proficient MSG speakers numbered no more than 30 in total, which has unfortunately declined since then. The average age of their informants was 75.2 years, further reinforcing the moribund condition of this dialect. Hopp and Putnam's (2015) aimed to test the robustness of asymmetric V2-patterns in MSG; i.e., whether or not the finite verb in matrix clauses generally appeared in second position and, conversely, as the final element in subordinate clauses (for an overview, see Vikner, 2020). Their study consisted of two components: (i) elicited semi-structured interviews with a researcher, and (ii) an acceptability judgment task (AJT). Although the speech of 14 participants were successfully recorded and analyzed in connection with the interviews, the number of those informants who were able to participate in the AJT was reduced by 43% (*n* = 8) due to a number of difficulties the informants encountered.

It would be counterproductive to draw these situations into the questions about the use and importance of “big data” in social science research because “big data” is often an unachievable standard based on the very nature of being a moribund language. Once again, such questions are misguided in this context if the research focus centers on gaining a better understanding of the underlying structural principles of a system of grammar according to a pre-established set of axioms and desiderata. It also cannot be overstated that findings from these studies whose findings are based on “limited data” can serve as the bedrock of further experimental work in other (heritage) bilingual populations, as in the case of the seminal research on Frenchville-French by Bullock and Gerfen (2004a,b), and Bullock and Gerfen (2005).

## 4. A Path Forward

The preceding discussion of the challenges linguists face when conducting research in these endangered HL-speaking communities must be counterbalanced with the importance of what this research can tell us about the development of bilingual grammars across the lifespan. In this sense, these toils and known difficulties are truly worth the effort. In this final section we outline a prudent and productive path forward for research on these grammars.

We ascertain that the most sensible and productive research focusing on these HLs grammars should first and foremost involve a detailed description of the extant (remaining) samples of these grammars. Such a strategy shares noticeable affinities with reconstruction techniques in descriptive and generative linguistics (Walkden, 2014), and specifically in efforts to reconstruct variation at shallow time depths (Labov, 1963; Janda and Joseph, 2003; Cravens, 2006; Geiger and Salmons, 2006; Lauersdorf, 2021). Although a particular analysis may only require a subset of data, that does not mean that these are the *only* valuable data. Furthermore, current understanding of what kinds of data are necessary and valuable may change in the future. This is all the more critical for moribund HLs, where we are approaching our final chances to collect and analyze what data there are available.

For moribund HLs, *all* of the data often amounts to fragmented sets or data drawn from a relatively few number of speakers. In such cases it is unclear how representative the data may be (or even if “representative” is an appropriate standard for a given community of moribund language speakers). Yet, these are the data that are necessary for establishing the basis for which a comparative or experimental study may proceed. Furthermore, these types of studies have their particular research questions, which are a subset of the questions linguistic scholars may pursue. An analysis of the American Norwegian patterns discussed in §2.2 does not necessarily require a comparison with English or a Norwegian baseline unless the research question motivates one, for example uncovering mechanisms for change in a heritage bilingual setting. The facts that [ɹ] is a possible outcome of AmNo retroflexion and that modified definite nouns primarily occur without an overt demonstrative warrant analyzes in and of themselves. A research question focused on uncovering the architecture and properties of those aspects of the AmNo grammar, or any HL grammar, does not require an appeal to how it may or may not differ from a monolingual comparison or align with the contacting language.

Like historical (socio)linguistics, we have to rely on descriptive and analytical tools that allow us to piece together a (more) complete picture from often limited and fragmentary data, along the lines of what Labov (1972, p. 100) describes as “the art of the historical linguist to make the best of this bad data.” Although we adamantly oppose the view that HL language data are in any sense “bad,” they are often not as rich or controlled as we might hope for (see §3), and accordingly we must triangulate them with available socio-historical information and context. And also like historical sociolinguists, moribund HL research draws on “the language data that history leaves us (what has “survived” through time), so [we must] wrap the language data in all possible socio-historical datasets to help complete the picture” (Lauersdorf, 2021, p. 216). Because comparative and experimental HL studies benefit from the completeness of that picture, it is incumbent upon us, as linguists, to consider the vast range of HL grammars and pursue the fullest possible descriptions of their forms and analyzes of their structures. The strength of any comparative study increases as a result of progress in that work. Ultimately, continued description and analysis of endangered languages, particularly among smaller communities, can only enrich our understanding of the properties of human language and help ensure that we do not avoid the pursuing the necessary questions for achieving that goal (see e.g., Leivada, 2021).

A full and comprehensive depiction of the development of bilingual grammars must include HL-speakers who speak an endangered, or moribund, vernacular (Putnam et al., 2018). These grammars are largely unaffected by any enforced prescriptive norms, which provide an opportunity for us, as linguists, to observe grammar development “in the wild.” However, as we have outlined in detail throughout this essay, the challenges associated with this research are sometimes quite daunting. The principle task at hand, at least at the initial stage of research on HL grammar, is one that is focused on *description*, and in some instances, *triangulation*. We agree with Bickel (2007, p. 242), who writes:

…extending our dataset by analyzing undescribed languages is not and should not be exclusively the task of typologists – especially not in these times of mass extinction of languages!

Although certainly appropriate for specific lines of inquiry (see Lohndal and Putnam, accepted), comparison of elements of HL-grammars in any attempt to establish the overall “complexity” of the system or degrees of distance between monolingual controls—or even other bi/multilingual populations—must be carried out under extreme caution in order to avoid an appeal to “deficit-based” bilingualism research (Ortega, 2014). It also cannot be overstated that any sort of comparison between HSs and another group is highly dependent on the specific nature of individual research questions and should not be the default *modus operandum* of this line of inquiry, and, as we have argued here, should only proceed once a detailed description of aspects of the grammar under investigation have been exhaustively described. In conclusion, research on moribund HLs has convincingly showed that, in spite of being acquired in third or fourth generation (and in rare cases, beyond the fourth generation), these HSs have retained a remarkable amount of grammatical complexity. The maintenance of highly functioning, grammatically complex systems extends even into the morphosyntax of receptive bilinguals (Sherkina-Lieber, 2015, 2020). In the years to come, we anticipate the number of receptive bilinguals to be on the rise, however, the research program we outline and advocate above is well-suited to include these individuals as we move forward.

## Author Contributions

All three authors (RD'A, DN, and MP) contributed equally to the research and writing associated with this article.

## Conflict of Interest

The authors declare that the research was conducted in the absence of any commercial or financial relationships that could be construed as a potential conflict of interest.

## Publisher's Note

All claims expressed in this article are solely those of the authors and do not necessarily represent those of their affiliated organizations, or those of the publisher, the editors and the reviewers. Any product that may be evaluated in this article, or claim that may be made by its manufacturer, is not guaranteed or endorsed by the publisher.

## Footnotes

^1^Of course, as pointed out to us by a reviewer, this quote in isolation is too strong and is slightly misleading. To clarify this main point of this quote, unmotivated comparisons of monolinguals and bilinguals generally lacks clear motivation, however, such a comparison is acceptable depending on the research questions pursued in individual studies. For example, in order to detect contact-induced language change, it is prudent to have a non-contact variety to function as a standard of comparison (or baseline).

^2^See also Ionin and Zyzik (2014) and Spinner and Gass (2019) for a discussion of the challenges of interpreting the results of judgment tasks in SLA research.
